# Assessment of *β*-D-glucosidase activity and *bgl* gene expression of *Oenococcus oeni* SD-2a

**DOI:** 10.1371/journal.pone.0240484

**Published:** 2020-10-09

**Authors:** Yahui Li, Ying Wang, Linlin Fan, Fan Wang, Xiaoli Liu, Hongzhi Zhang, Jianzhong Zhou

**Affiliations:** 1 Institute of Agro-product Processing, Jiangsu Academy of Agricultural Sciences, Nanjing, Jiangsu, China; 2 School of Food and Biological Engineering, Jiangsu University, Zhenjiang, China; Murdoch University, AUSTRALIA

## Abstract

Glycosidases enhance flavor during wine-making by mediating the enzymatic release of aroma molecules. In order to better understand the aroma enhancement potential of *Oenococcus oeni* SD-2a, *β*-D-glucosidase (*β*G) activities in the culture supernatant, whole cells, and disrupted cell lysate were assessed at mid log, late log and stationary growth phase. The enzymatic activity was also compared further from cell cultures with 5 different carbon sources (glucose, cellobiose, arbutin, glucose and cellobiose, glucose and arbutin) at late log phase. Correspondingly, expression levels of 3 *bgl* genes, *OEOE-0224*, *OEOE-1210*, and *OEOE-1569* were investigated from cell cultures of the 3 growth phases, and the 5 cell cultures with different carbon sources. Finally, the volatile aroma compounds released by *O*. *oeni* SD-2a in synthetic wines with natural glycosides were evaluated by GC-MS. Results showed *β*G of *O*. *oeni* SD-2a was not extracellular enzyme, and the location of it didn’t change with the change of growth phase and carbon source studied. *β*G activities in the whole cells and disrupted cell lysate were similar and constant at the 3 growth phases. As for the carbon sources, *β*G activities of whole cells and disrupted lysate were positively affected by cellobiose. While arbutin displayed positive and negative effect on *β*G activity of whole cells and disrupted lysate, respectively. It is probably that *bgl* genes *OEOE-0224* and *OEOE-1210* were related to *β*G activity of SD-2a whole cells, while *OEOE-1569* was responsible for *β*G activity of disrupted lysate. More kinds of volatile compounds and higher total concentration were released by SD-2a in synthetic wine compared with control. Thus, SD-2a showed a great potential for flavor enhancement under wine-like conditions. This study provides more information for further study of *β*G activity from *O*. *oeni* SD-2a.

## Introduction

Most white wines and almost all red wines undergo a secondary fermentation, or malolactic fermentation (MLF), a process in which dicarboxylic L-malic acid is decarboxylated into monocarboxylic L-lactic acid [1,2]. This bioconversion is carried out by lactic acid bacteria (LAB), especially by *Oenococcus oeni* the predominant LAB to conduct MLF [3]. In addition to decreasing wine sourness and increasing wine microbial stability via the metabolism of L-malic acid, MLF can also lead to flavor modification through the bacterial production of various secondary metabolites [4–6].

Aroma compounds present in grape and wine include volatile aroma substances, especially terpenes, which play a fundamental role in wine flavor, and aroma precursors, which are odorless, but could produce volatile aroma after hydrolysis [7–9]. These odorless precursors are largely present in grape and wine as glycosides, often structured as mono-glucoside in which the volatile aglycone is bound to a glucose moiety, or as disaccharide in which the glucose moiety is further substituted with a second sugar such as *α*-L-arabinopyranose, *α*-L-rhamnopyranose, *β*-D-apiofuranose and *β*-D-xylopyranose [10]. Volatile aglycones in these glycosides constituting a reserve of powerful odor active-compounds are often dominated by monoterpenes, C_13_-norisoprenoids and benzene derivatives and can greatly enhance wine aroma after release from glycosides [2,8,10].

These bound volatile aglycones are released either by acid hydrolysis, or by the action of glycosidase [8,11]. Acid hydrolysis, occurring slowly during wine storage, may produce undesirable and unpredictable flavors [1,8,12]. Alternatively, enzymatic hydrolysis is a favorable method to enhance the natural aroma spectrum of wine without detrimental effects on wine quality [13]. Thus, it is widely used during winemaking nowadays.

*β*-D-Glucosidase (*β*G) is one important glycosidase to hydrolyze glycosylated aroma precursors, releasing active aroma and flavor compounds during winemaking [14]. Typically, volatile aglycones present in mono-glucoside are directly released via *β*G, while diglycoside-bound aglycones are sequentially released by *α*-L-arabinopyranosidase, *α*-L-rhamnopyranosidase, *β*-D-apiofuranosidase, or *β*-D-xylopyranosidase followed by *β*G [9,14]. Over the past decades, an increased interest in the sources of *β*G during winemaking had been focused on LAB, especially *O*. *oeni* strains. Numerous studies had been conducted, providing evidence of the potential *β*G activity of *O*. *oeni* strains for flavor enhancement in wines [7,10,14–24]. These studies demonstrated that possession of *β*G activity is widespread and extent of *β*G activity is strain dependent among *O*. *oeni* strains [16,21,25,26], and the enzyme activity is mainly affected by pH, temperature, sugar, and ethanol concentration [15–17,19,27,28].

*O*. *oeni* SD-2a is one patent strain screened from spontaneous MLF wines in Yantai, Shandong Province, China, which with an excellent performance in MLF is widely used in winemaking. In previous study, *β*G activity of *O*. *oeni* SD-2a was localized, assessed with synthetic and natural substrates and partially characterized under different physicochemical conditions and abiotic stresses [11,26,29]. Also, one *bgl* gene of *O*. *oeni* SD-2a was cloned, sequenced and analyzed [24]. In order to better understand the potential ability of *O*. *oeni* SD-2a to enhance wine aroma and flavor during fermentation, in this study, *β*G activity of *O*. *oeni* SD-2a was further evaluated with synthetic and natural substrates, also expression levels of different *bgl* genes were investigated.

## Materials and methods

### Bacterial strains and cultivation

*O*. *oeni* SD-2a, stored in our laboratory, was used in this study. The strain was cultivated as described before [29]. Bacterial cultures were prepared by inoculating 1.0% (v/v) of pre-cultures into acidic tomato broth (ATB) medium and incubated at 25°C. Growth of *O*. *oeni* SD-2a was monitored by OD_600nm_. ATB medium containing: glucose10 g/L, yeast extract 5 g/L, peptone 10 g/L, MgSO_4_∙7H_2_O 0.2 g/L, MnSO_4_∙4H_2_O 0.05 g/L, cysteine/HCl 0.5 g/L, and tomato juice 250 mL/L. pH was adjusted to 4.8 with KOH.

### Sample preparation

#### Samples at different growth phases

*O*. *oeni* SD-2a cultures were prepared in ATB medium and sampled at mid log (48 h), late log (80 h) and stationary phase (98 h) of growth for enzyme activity assay and RNA extraction. Samples were prepared in triplicate.

#### Samples with different carbon sources

Carbon source in ATB medium 10 g/L glucose was replaced as 10 g/L cellobiose, 10 g/L arbutin, 5 g/L glucose and 5 g/L cellobiose, or 5 g/L glucose and 5 g/L arbutin, to get another 4 mediums with different carbon sources. *O*. *oeni* SD-2a cultures were prepared in these 5 mediums and sampled after 80 h for enzyme activity assay and RNA extraction. Samples were prepared in triplicate.

### Sample treatment and enzyme assay

Sample treatment was conducted according to the method described previously with some modifications [11]. In brief, 1 mL of culture was centrifuged (5,000×g, 4 °C for 10 min) to collect whole cells and supernatant. Whole cells were washed with 1 mL 150 mmol/L NaCl, then re-suspended in 1 mL cold aseptic water for enzyme assay. Supernatant was directly used to determine enzyme activity. For intracellular enzyme assay, washed whole cells were re-suspended in 1 mL phosphate buffer saline (PBS) 1 x buffer (0.01M, pH = 7.2), sonicated in an ice bath using a sonicator (Hielscher GmbH, Germany) at 100 w for 20 min, then disrupted lysate was used for enzyme assay. *β*G activity assay was conducted following the method described by Li et al. [11]. One unit of enzyme activity was defined as *u*mol of *p*-nitrophenol (*p*-NP) released per min per gram of dry cell weight.

### RNA extraction

*O*. *oeni* SD-2a cells were harvested by centrifugation (5,000×g, 4 °C for 10 min).Total RNAs was extracted according to the instructions of the RNAisoPlus kit (Takara, Dalian, China). RNA concentration and quality were evaluated by measuring absorbance at 260 nm and 280 nm using the NanoDrop ND-2000 Spectrophotometer (Thermo Fisher, Waltham, MA, USA) and by analysis on a 1.0% (w/v) agarose gel.

### *bgl* genes sequences and primers design

Nucleotide sequences of *O*. *oeni* strain PSU-1 were obtained from the National Center for Biotechnology Information (NCBI) web site (www.ncbi.nlm.nih.gov). The sequence references of glucosidase genes from *O*. *oeni* PSU-1 (NC_008528) were as follows: *bgl OEOE-0224* (ID:4416011) and *OEOE-1210* (ID:4415643) coding for 6-phospho-*β*-glucosidase (EC: 3.2.1.86), while *bgl OEOE-1569* (ID:4416192) coding for *β*-glucosidase (EC: 3.2.1.21).

Primers for real-time qPCR were designed using Primer3 software and checked for gene-specific binding using NCBI primer design tool (http://www.ncbi.nlm.nih.gov/tools/primer-blast/). Gene *ldhD* and gene *pta* were used as the internal standard [1,30,31]. All primers in Table 1 were purchased from Sangon-Biotech (Shanghai, China).

**Table 1:** Primers used for real time-qPCR.

Gene	Primers (5′–3′)		Amplicon length (bp)
*OEOE-1569*	1569 F	AAGTCCTGTTTCCTTTTGGTCA	195 bp
	1569 R	TTAGTTCCTTCAGCGGTTTTTC	
*OEOE-1210*	1210 F	AGCGGTCCTGATAATTCTGATTG	150 bp
	1210 R	GAAATCCCCTTCACCTTCTGG	
*OEOE-0224*	0224 F	GACGAGGATCTTTCCCAATG	102 bp
	0224 R	GGTTCGATACCGTACTTGTG	
*ldhD*	ldhD F	GCCGCAGTAAAGAACTTGATG	102 bp
	ldhD R	TGCCGACAACACCAACTGTTT	
*pta*	pta F	CATGGCTGAGATTGCCGTTC	149 bp
	pta R	TCTCCTGCGCCAGCTTAGT	

### Reverse transcription and real-time qPCR

cDNA was synthesized with 200 ng/μL RNAs using the Primer Script RT reagent Kit with gDNA Eraser (Takara, Dalian, China) as recommended. Real-time qPCR was performed using the CFX96 Touch (Bio-Rad, Hercules, CA, USA) and following the manufacturer of SYBR Premix ExTaq II kit (Takara, Dalian, China). Thermal cycling conditions were as follows: an initial step at 95°C for 30 s, followed by 40 cycles of 95°C for 5 s and 60°C for 30 s. A melting curve was established in the end of the run and efficiency of amplifications was determined by a standard curve. In each run, a negative control was included. The relative gene expression was calculated using threshold cycles by the 2^−ΔΔCT^ method, with internal control genes *ldhD* and *pta* for normalization. The sample at mid log phase (48 h) of growth and sample cultured with glucose in ATB medium were used as reference sample, respectively, which represented 1× expression of the target gene.

### Preparation of aroma precursors

The precursors were extracted from the grape variety of Chardonnay cultivated in Shaanxi province, China. The procedure was based on the method described by others [10]. The final dried extract from 1000 g grapes was dissolved in 20 mL citrate-phosphate buffer (1M, pH 5.0) and stored at -20°C.

### Fermentation in synthetic wine

Malolactic fermentation was carried out in 1L synthetic wine containing nutrients and other bacteria requirements. The composition of this growth medium was similar to that reported [32], except that aroma precursors extract was added at the amount of 20 mL/L (equivalent to an aliquot of glycosides from 1000 g grapes) and pH was adjusted to 3.6 with KOH pellets. The synthetic wine was sterilized by 0.22 *μ*m membrane filtration, and inoculated with 1.0% (v/v) of *O*. *oeni* SD-2a cultures previously grown at ATB medium after 48 h. A non-inoculated reference sample was prepared by adding 1.0% (v/v) of sterile water to the synthetic wine. All samples were carried out in triplicate and incubated at 25°C. The evolution of MLF was monitored by measuring the content of L-malic acid using an enzymatic kit (Megazyme, Ireland). MLF was inhibited by means of 50 mg/L of sulfur dioxide, when the content of malic acid was lower than 0.2 g/L.

### Aroma analysis by gas chromatography-mass spectrometry (GC-MS)

The volatile aroma compounds of samples treated above were monitored by GC-MS. The GC-MS conditions and analysis followed the method described with some modifications [33]. GC-MS analysis was performed using a 7890A GC, equipped with a HP-INNOWax capillary column (0.25 mm × 30 m × 0.25 *μ*m), coupled with HP 5975C mass spectrometer and 7693A automatic injector (Agilent, Santa Clara, CA, USA). Oven temperature program: after 5 min at 50 °C, heating 5 °C/min until 150 °C, then 5 °C/min until 220 °C, finally 220 °C isothermal for 10 min. The mass parameters: electron—70 eV, an ion source temperature of 230 °C, and a quadrupole temperature of 150 °C with a mass range of m/z 33–600. Other experimental conditions: injector temperature 230 °C, helium at flow rate of 1.0 mL/min, sample volume injected 1 *μ*L. All samples were directly injected, with no dilution. Identification of volatile compounds was carried out by comparing them with the retention times of standards and with the NIST Mass (rev08) Spectral Database. For other compounds the retention index was calculated by using a standard mixture of C_11_−C_32_ aliphatic hydrocarbons. The concentration of compounds was quantified as *μ*g/L using relative areas related to the internal standard 1-decanol.

### Statistical analysis

The mean values of those triplicated counts were subjected to analysis of variance using the SAS statistical software (SAS Institute, Cary, USA) at the 1% level of significance.

## Results

### Enzyme assay at different growth phases

**Fig 1:**
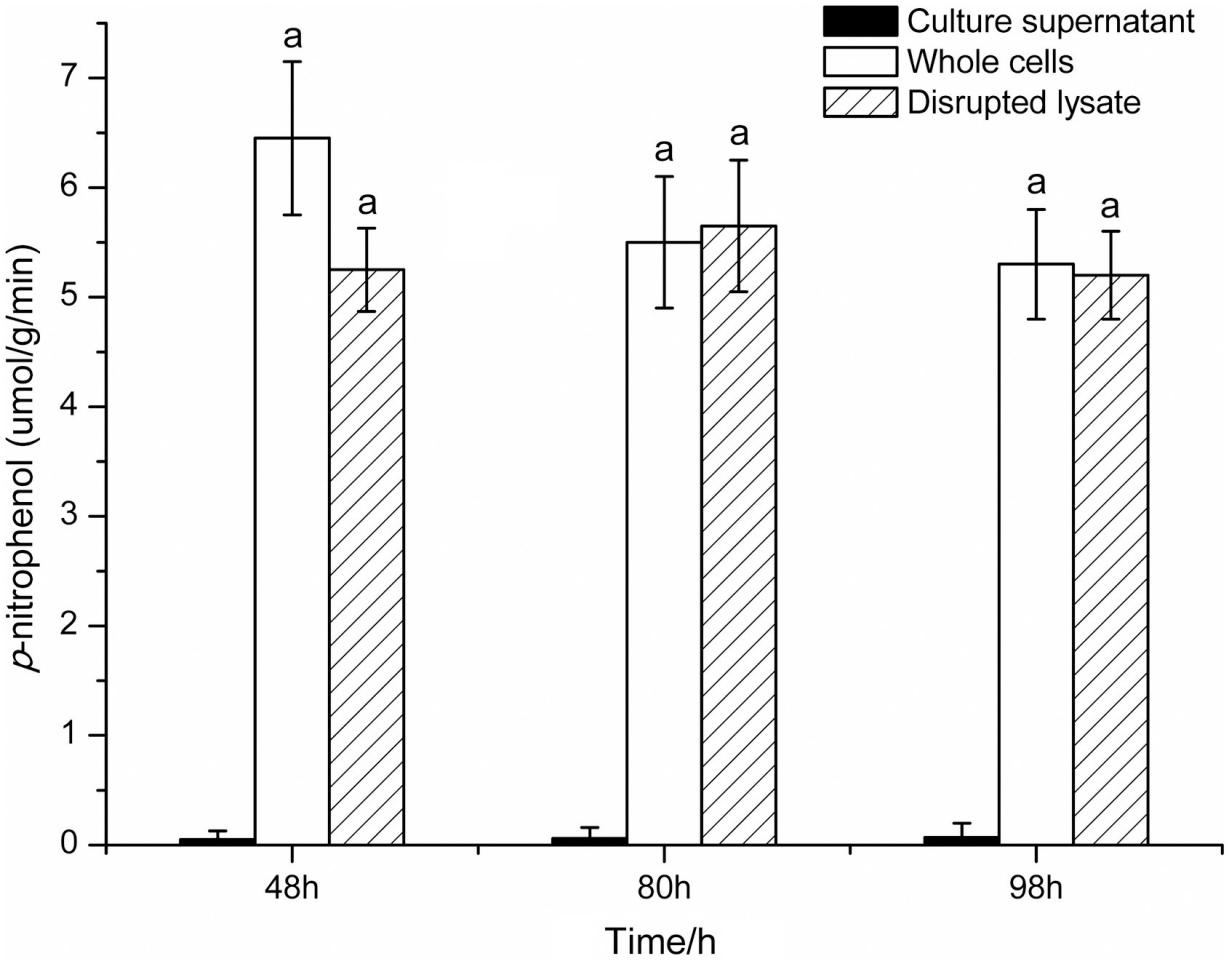
*β*-D-glucosidase activities of *O*. *oeni* SD-2a at different growth phases.

*β*G activities in culture supernatant, whole cells, and disrupted lysate of *O*. *oeni* SD-2a were assessed at mid log (48h), late log (80h), and stationary phase (98h) of growth. As shown in Fig 1, almost no activities were detected in culture supernatant, while obvious activities were observed in whole cells and disrupted lysate at all growth phases tested. No significant differences were observed among whole cells and disrupted lysate at each growth phase (*P*<0.05), also between different growth phases for both whole cells and disrupted lysate. These are consistent with the report about *β*G localization in the same strain [11]. This indicates no *β*G was secreted outside the cells, and *β*G activities in whole cells and disrupted lysate were similar and constant during the growth of *O*. *oeni* SD-2a.

### Enzyme assay with different carbon sources

**Fig 2:**
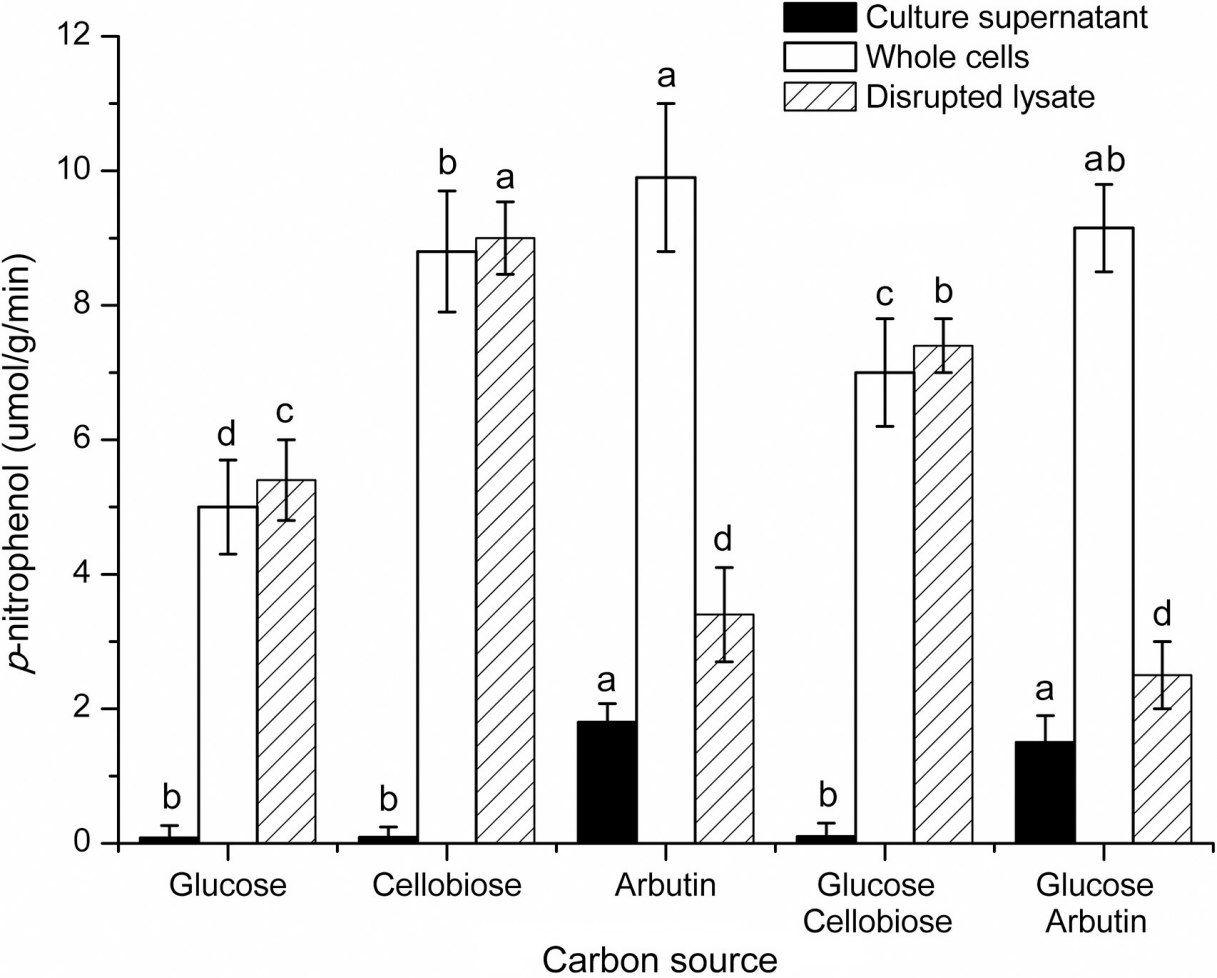
*β*-D-glucosidase activities of *O*. *oeni* SD-2a cultured with different carbon sources.

*β*G activities in culture supernatant, whole cells, and disrupted lysate of *O*. *oeni* SD-2a were also measured after SD-2a was cultured with different carbon sources (Fig 2). The enzyme activities varied greatly when SD-2a was cultured with glucose, cellobiose, arbutin and their mixtures individually. Obvious *β*G activities were detected in culture supernatant with the carbon source containing arbutin, while fairly low activities with glucose, cellobiose and their mixture. This may indicate that *β*G was secreted into the medium under the induction of arbutin. For whole cells, higher enzyme activities were observed with carbon source containing cellobiose and arbutin, especially containing arbutin, than that with glucose. Moreover, whole cells showed higher enzyme acidities with cellobiose and arbutin than that with their mixture with glucose correspondingly. This indicates that carbon source cellobiose and arbutin can significantly increase *β*G activity of whole cells of *O*. *oeni* SD-2a. As for disrupted lysate, *β*G activities varied obviously under the induction of different carbon sources. Enzyme activities increased with carbon source containing cellobiose, while decreased with carbon source containing arbutin, compared with that with glucose. It should be noted that *β*G activities in disrupted lysate were significantly lower than that in whole cells with carbon source containing arbutin, while no significant differences were observed between them with other carbon sources (*P*<0.05). This is the first time to describe the influence of carbon source on *β*G activity of *O*. *oeni* strains. Similar report has not been reported before.

### Gene expression at different growth phases

**Fig 3:**
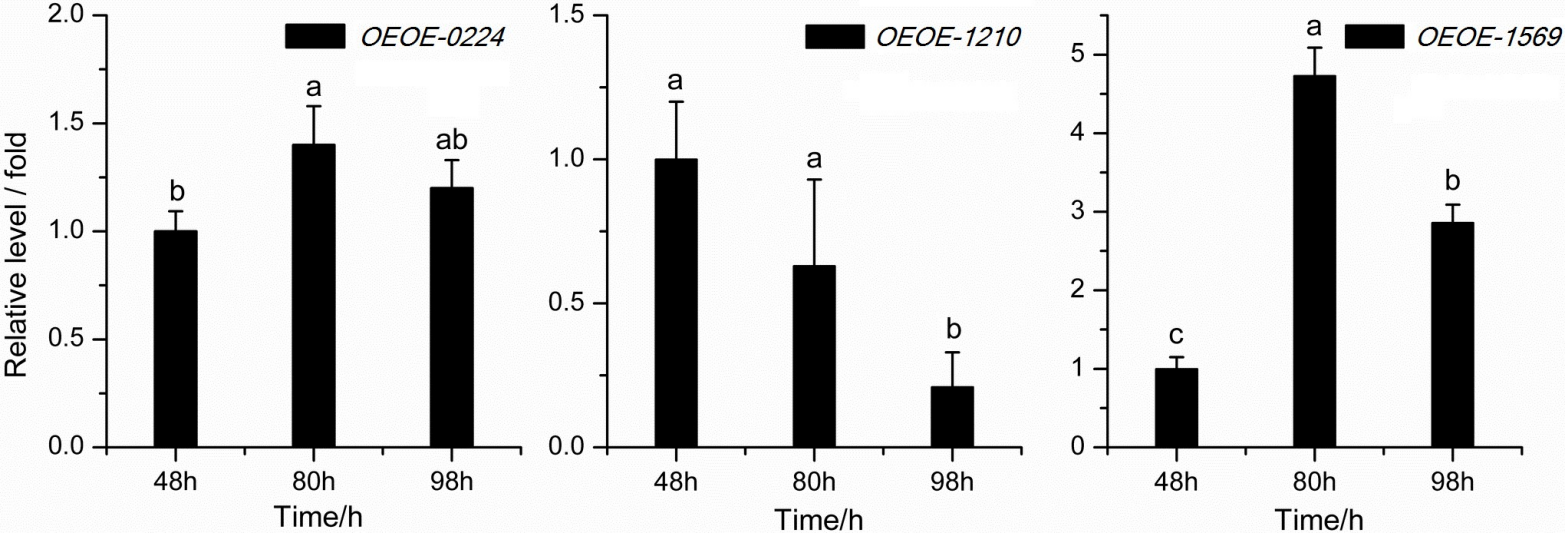
Expression levels of *bgl* gene in *O*. *oeni* SD-2a at different growth phase. Note: The sample at 48h represented 1× expression of target gene.

The expression levels of *bgl* gene *OEOE-0224*, *OEOE-1210*, and *OEOE-1569* in *O*. *oeni* SD-2a were conducted at different growth phases, corresponding to enzyme assay at different growth phases (Fig 3). The sample at 48h was used as reference, which represented 1× expression of target gene. The expression levels of gene *OEOE-0224* increased at 80h and decreased at 98h, compared with that at 48h, but no significant difference between them. Similar results have been described in *O*. *oeni* PSU-1 and 217 [1]. For gene *OEOE-1210*, it was down regulated with growth time. Significant increase was observed for the third gene *OEOE-1569*, with expression level higher at 80h than that at 98h. *OEOE-1569* showed the greatest change in expression level with growth of SD-2a in the 3 genes.

### Gene expression with different carbon sources

**Fig 4:**
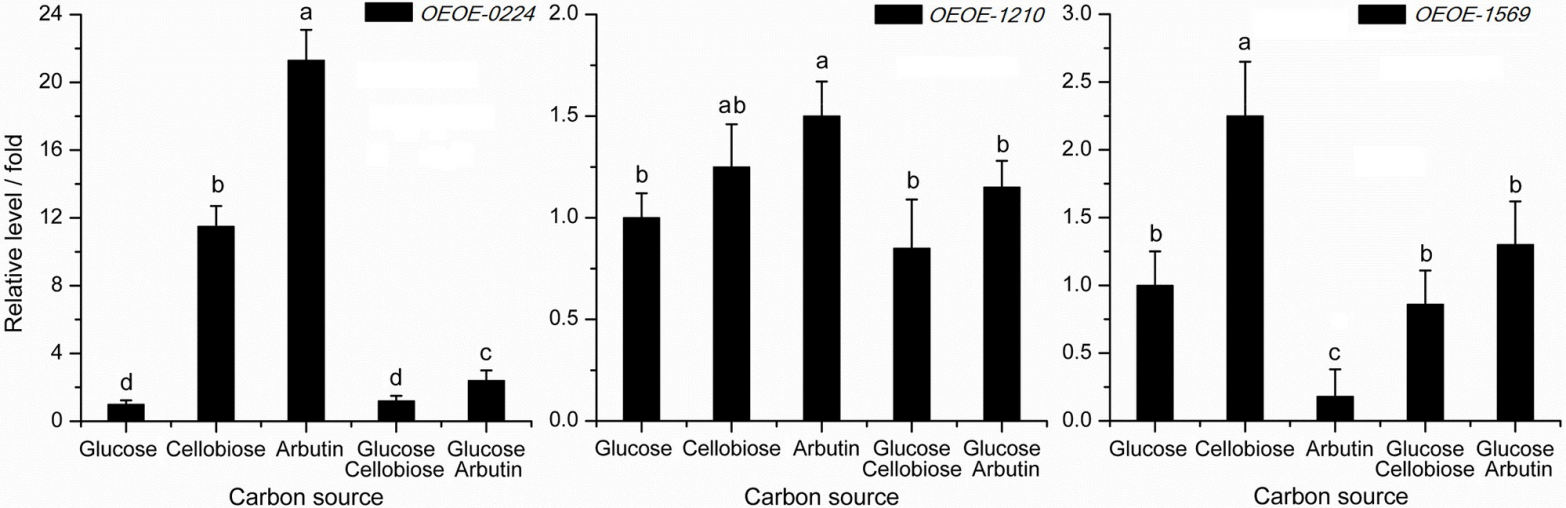
Expression levels of *bgl* gene in *O*. *oeni* SD-2a cultured with different carbon sources. Note: The sample cultured with glucose represented 1× expression of target gene.

The expression levels of the 3 *bgl* genes were also evaluated after *O*. *oeni* SD-2a was cultured with different carbon sources, corresponding to enzyme assay with different carbon sources (Fig 4). The sample cultured with carbon source glucose was used as reference, which represented 1× expression of target gene. As shown in Fig 4, the greatest variation in expression level was observed in *OEOE-0224*. It increased significantly with carbon sources arbutin, cellobiose and the mixture of glucose and arbutin, especially with arbutin and cellobiose of 21.3 folds and 11.5 folds, respectively. For gene *OEOE-1210*, higher expression levels were also observed with carbon sources arbutin, cellobiose and the mixture of glucose and arbutin, but no significant difference compared with that with glucose, except that with arbutin. As for *OEOE-1569*, obvious increase in expression level was detected with cellobiose, while down-regulation with arbutin. Influence of carbon source on the expression level of 3 *bgl* genes has not been reported before.

### Aroma released from natural precursors in synthetic wine

Table 2 shows the changes of volatile compounds profile in a wine-like medium supplemented with glycosides after MLF carried out by *O*. *oeni* SD-2a. Un-inoculated medium as reference was also evaluated. Totally, 16 compounds with a total concentration of 222.51 *μ*g/L were identified in reference, while 23 compounds with a total concentration of 449.18 *μ*g/L in sample with SD-2a. Another 10 new compounds were detected in SD-2a sample compared with that in reference. Most of these compounds were previously identified in Chardonnay juice or wine [34–36]. Compounds identified in reference mainly included acids, alcohols, terpenes and esters in total concentrations of 64.95, 46.77, 39.12 and 34.91 μg/L individually, and in minor proportion benzenic compounds and other compounds. Decanoic acid, 1-hexanol, alpha-terpineol and diethyl monosuccinate were the most abundant volatile compounds in the pools of acids, alcohols, terpenes and esters. In the sample with SD-2a, total concentrations of esters, terpenes and acids increased by up to 441%, 173% and 61% individually, while alcohols, benzenic compounds and other compounds decreased 31%, 75% and 31% individually, compared with that in reference. Theses significant increases were mainly due to the formation of new compounds such as ethyl dodecanoate, ethyl lactate, methyl vanillate, linalool, citronellal, octanoic acid, hexanoic acid and the increment of ethyl octanoate, diethyl succinate, alpha-terpineol and 4-hydroxybenzoic acid. The increment of esters was much higher than that of terpenes and acids. Methyl vanillate (83.04 μg/L), ethyl lactate (61.94 μg/L) and ethyl octanoate (31.38 μg/L) in esters, alpha-terpineol (58.42 μg/L) and citronellal (40.82 μg/L) in terpenes and 4-hydroxybenzoic acid (56.18 μg/L) in acids were the most abundant volatile compounds detected in SD-2a sample. The concentrations of alcohols and benzenic compounds were negatively affected by SD-2a, however, new formations of 3-methyl-1-butanol and benzyl alcohol were detected. In addition, the concentration of 3-hydroxydamascone increased, furfural formed, while 3-methylbutanal disappeared. The composition of volatile compounds in reference remained almost unchanged, confirming that all changes in sample were caused by the growth and metabolism of *O*. *oeni* SD-2a.

**Table 2:** Aglycones released from natural precursors by *O*. *oeni* SD-2a in synthetic wine after MLF.

Aglycons		Concentration (μg/L)	
		Reference	SD-2a
Esters	Ethyl octanoate	2.52±0.18	31.38±2.85
	Ethyl dodecanoate	ND	2.56±0.21
	Ethyl lactate	ND	61.94±3.21
	Diethyl monosuccinate	22.45±1.02	4.65±0.23
	Methyl vanillate	ND	83.04±3.89
	Diethyl succinate	1.58±0.09	3.86±0.28
	Diethyl malate	8.36±0.12	1.59±0.03
Total		34.91	189.02
Terpenes	Alpha-terpineol	18.73±0.68	58.42±2.54
	Linalool	ND	1.43±0.19
	Geraniol	12.23±0.70	ND
	Citronellal	ND	40.82±2.15
	Limonene	8.16±0.12	6.34±0.57
Total		39.12	107.01
Acids	4-hydroxybenzoic acid	23.45±1.20	56.18±4.35
	Octanoic acid	ND	2.47±0.05
	Hexanoic acid	ND	20.68±1.03
	Decanoic acid	32.86±1.25	24.26±2.01
	Nonanoic acid	8.64±0.04	0.97±0.07
Total		64.95	104.56
Alcohols	1-hexanol	35.25±2.15	23.54±1.89
	2-phenylethanol	11.52±0.52	2.68±0.23
	3-methyl-1-butanol	ND	5.84±0.42
Total		46.77	32.06
Benzenic compounds	Benzyl alcohol	ND	4.02±0.24
	Phenylethyl alcohol	5.62±0.49	0.89±0.08
	4-vinylguaiacol	14.21±1.30	ND
Total		19.83	4.91
Other	3-hydroxydamascone	4.26±0.34	10.73±1.12
	Furfural	ND	0.89±0.09
	3-methylbutanal	12.67±0.53	ND
Total		16.93	11.62
**Total**		**222.51**	**449.18**

Note: The data is mean value of triplicate samples (maximum SD:±10%); ND, Not determined.

## Discussion

Research in progress shows that LAB, possessing glycosidase activities, can modify sensory properties and aroma profile of wine. Glycosidase activities are of interest as they are responsible for the release of volatile aromas in winemaking [2,37]. Mostly, attentions are focused on *β*G activity of *O*. *oeni* strains during MLF [2,16,38]. *O*. *oeni* SD-2a, one patent strain screened in China, possesses important oenological characteristics, particularly being able to perform MLF effectively under winery conditions. In previous study, *β*G activity of *O*. *oeni* SD-2a was assessed and partially characterized [11,26,29]. In order to enable a better understanding of the capacity of *O*. *oeni* SD-2a to aroma formation during MLF. In present study, *β*G activity of SD-2a was evaluated at different growth phases and after SD-2a was cultured with different carbon sources with synthetic substrate. To find the key *β*G gene for enzyme activity, expression levels of 3 *bgl* genes were also investigated corresponding to the enzyme assay. Finally, influence of *O*. *oeni* SD-2a on the changes of volatile compounds in wine-like medium supplemented with glycosides extract was conducted after MLF.

Localization of *β*G showed *β*G in *O*. *oeni* SD-2a was intracellular form, and whole cells of SD-2a possessed almost the same enzyme activity as total activity [11]. To figure out whether and how the location and activity of *β*G in *O*. *oeni* SD-2a vary during growth, *β*G activities in culture supernatant, whole cells and disrupted lysate of SD-2a were evaluated at mid log, late log and stationary phase of growth in present study. Results showed few activities in culture supernatant and almost same activities in whole cells and disrupted lysate at all phases studied. This indicated that no *β*G was secreted into the medium, the location of *β*G in *O*. *oeni* SD-2a didn’t change with growth, and total activity and enzyme activity in whole cells were similar and constant during growth. However, this ran contrary to the report about *β*G activity of some commercial *O*. *oeni* strains. In the report, *β*G activity occurred in both supernatant and whole cells and varied according to the growth stage, with a high change in distribution of *β*G activity between supernatant and whole cells [39]. Intracellular and extracellular *β*G activity was also reported in other *O*. *oeni* strains [20,40,41]. This seems to suggest that the location of *β*G in *O*. *oeni* strains is variable and strain dependent. It is worth noting that considerable enzyme activity was detected in whole cells of SD-2a, also in whole cells of other *O*. *oeni* strains [13,16,20]. This is of great significance, since whole cells could be directly utilized in application.

It is reported that the presence of preferred sugar, such as glucose, represses the synthesis of some enzymes in microbes, which are responsible for the metabolism of less favorable carbon source, such as glycosides. This phenomenon became known as glucose effect or carbon catabolite repression (CCR) [42]. According to CCR, when the preferred carbon source is absent or exhausted, enzymes that metabolize the non-preferred carbon source producing monosaccharides will be synthesized, such as translocase and glycosidase. Induction of *β*G activity by glucosides in sugar depleted medium has been reported in *S*. *cerevisiae* [43]. In present study, *β*G activities in culture supernatant, whole cells, and disrupted lysate of *O*. *oeni* SD-2a were investigated after SD-2a was cultured with preferred and non-preferred carbon sources respectively. Results showed whole cells and disrupted lysate of SD-2a possessed *β*G activities with all carbon sources studied and enzyme activities varied with different carbon sources. So the *β*G in SD-2a was probably constitutive enzyme, not inducible. It is noteworthy that obvious activities were observed in culture supernatant with carbon source containing arbutin. This may be caused by *p*-hydroxyphenol released from arbutin, since *p*-hydroxyphenol being same as *p*-nitrophenol, produced a yellow color under alkaline condition in enzyme assay. This has been confirmed in subsequent study (data is not shown). Thus, it follows that *β*G was not extracellular when SD-2a was cultured with different carbon sources studied. This, consistent with the location of *β*G in SD-2a [11], suggests location of *β*G in SD-2a didn’t change with the change of carbon source. As for disrupted lysate, *β*G activity with carbon source cellobiose was significantly higher than that with glucose. This is in agreement with CCR theory. However, it is contrary to CCR theory with arbutin, with the *β*G activity lower than that with glucose. Generally, in order to obtain enough glucose during growth, bacteria will show higher *β*G activity when using arbutin as carbon source, compared with using cellobiose, as two glucoses could be produced after degradation of cellobiose, while only one for arbutin. However, opposite results were observed in this study. The reasons for this are under investigation. Interestingly, the *β*G activity of whole cells of SD-2a coincided well with the CCR theory and inference. Higher *β*G activities were observed with carbon sources cellobiose and arbutin than that with glucose, with the highest activity with arbutin. Moreover, significant differences were observed in *β*G activities between whole cells and disrupted lysate when SD-2a was cultured with carbon source containing arbutin, although no differences with other carbon sources studied. These differences in *β*G activities between whole cells and disrupted lysate may be caused by different glucosidase responsible for them. *β*G activity of whole cells could be explained by the presence of 6-phospho-*β*G in SD-2a, which is the important component of phosphoenolpyruvate dependent phosphotransferase system (PEP-PTS) in bacteria [3], while *β*G activity of disrupted lysate may be due to the intracellular *β*G in SD-2a. 6-phospho-*β*G is one different glucosidase from *β*G and degrades phosphorylated substrates specifically. PEP-PTS is a bacterial transport system that allows bacterial cells to grow on various carbon sources, including glucosides [42]. It is reported that *O*. *oeni* strains was able to take up glucosides via the PEP-PTS, involving phosphorylation and the subsequent hydrolysis of phosphorylated glucosides in the cytoplasm through the action of 6-phospho-*β*G [44]. This hypothesis could well explain the phenomenon that whole cells of *O*. *oeni* strains showed high *β*G activity, while low or no intracellular and extracellular *β*G activities observed [11,13,29,45].

In total, 6 *bgl* genes have been reported in *O*. *oeni* strains, which are AG1, ORF1 (*OEOE-0224*), ORF2 (*OEOE-0341*), ORF3 (*OEOE-0340*), ORF4 (*OEOE-1210*) and ORF5 (*OEOE-1569*) [46]. Of these, AG1, ORF1, ORF2, ORF3 and ORF4 share homology to glycosyl hydrolase family (GHF) 1, coding for 6-phospho-*β*G, while ORF5 shows high homology to GHF3, coding for *β*G. In order to verify the hypothesis and find the gene responsible for *β*G activity of SD-2a whole cells, expression levels of different *bgl* genes in *O*. *oeni* SD-2a were conducted at the same time of enzyme assay. AG1 at the location of 211150–212482 is part of *OEOE-0224* with the location of 211150–212592. No effective primers were found for *OEOE-0340* and *OEOE-0341*, maybe due to the low expression. Thus, in this paper, another three *bgl* genes *OEOE-0224*, *OEOE-1210*, and *OEOE-1569* were studied. No similar trend was observed between the enzyme activities and gene expressions by comparing Figs 1 and 3. However, in Figs 2 and 4, expression levels of *OEOE-0224* and *OEOE-1210* showed the same trend with *β*G activities of SD-2a whole cells, and expression levels of *OEOE-1569* showed the similar trend with *β*G activities of disrupted lysate. This to a certain extent confirms the hypothesis and suggests that *bgl* genes *OEOE-0224* and *OEOE-1210* are related to *β*G activity of SD-2a whole cells and *OEOE-1569* are responsible for *β*G activity of disrupted lysate. Up to now, only a few studies on *bgl* genes of *O*. *oeni* strains have been reported [1,3,18,44], and expression of these genes has not been reported before, except *OEOE-0224* [1]. Further study focusing on which one of the 6 *bgl* genes is mainly responsible for *β*G activity of SD-2a whole cells is ongoing.

To give more light on the aromatic potential of *O*. *oeni* SD-2a during MLF, the changes in volatile compounds profile were determined in synthetic wine supplemented with natural precursors at the end of MLF. Some researchers have pointed out that glycosidase assay depends to a large extent on the chemical structures of the substrate, and natural aroma precursors are necessary for an adequate evaluation of the glycosidase potential of *O*. *oeni* strains [19]. So natural glycosides extracted from Chardonnay were used in this study. The ability of *O*. *oeni* SD-2a to degrade glycosides was investigated in citrate-phosphate buffer in our previous study [26], while in present study it is tested in synthetic wine, which is close to the wine. Totally 10 more volatile compounds and 102.09 *μ*g/L higher in total concentration were released by SD-2a in synthetic wine in present study, compared with that in citrate-phosphate buffer in previous study, with the same glycoside extract. It follows that environment may have a big influence on glycosidase activities of SD-2a in practice. Thus evaluation in wine is necessary in the future. Another 10 new volatile compounds and 226.67 *μ*g/L higher in total concentration were detected in sample with SD-2a than that in reference in present study. This may indicate that *O*. *oeni* SD-2a shows positive effect on aroma formation and proves promising for flavor enhancement during winemaking. In fact, it is not accurate to evaluate the ability of *O*. *oeni* strains to degrade glycosides and produce volatiles only by *β*G activity. As most natural glycosides, existing in the form of disaccharides, degrade sequentially via *α*-L-arabinopyranosidase, *α*-L-rhamnopyranosidase, *β*-D-apiofuranosidase, or *β*-D-xylopyranosidase followed by *β*G [2]. So far, numerous glycosidases have been reported in *O*. *oeni* strains [47]. So assessment of other glycosidases activities of *O*. *oeni* SD-2a is required in further research.

## Conclusions

*β*G of *O*. *oeni* SD-2a was not extracellular and not inducible enzyme, and the location of it didn’t change with the change of growth phase and carbon source studied. Whole cells and disrupted lysate of SD-2a possessed significant *β*G activities with the carbon source glucose and they were similar and constant during the growth. Non-preferred carbon sources cellobiose showed a positive effect on *β*G activities of whole cells and disrupted lysate compared with preferred carbon source glucose. While arbutin displayed a positive and negative effect on *β*G activities of whole cells and disrupted lysate, respectively. Expression levels of 3 *bgl* genes varied greatly with the change of growth phase and carbon source. It is probably that *bgl* gene *OEOE-0224* and *OEOE-1210* were related to *β*G activity of SD-2a whole cells, while *OEOE-1569* was responsible for *β*G activity of disrupted lysate, through the comparison of enzyme activities and expression levels of *bgl* gene. SD-2a, showing a great potential for flavor enhancement under the wine-like conditions, could be of great interest for its utilization in MLF. This study provides more information about *β*G of *O*. *oeni* SD-2a, and contributes to the preliminary knowledge of it for further study. Clarifying which *bgl* gene is mainly responsible for *β*G activity of *O*. *oeni* SD-2a whole cells will be conducted in the future.
